# Reduced genetic diversity associated with the northern expansion of an amphibian species with high habitat specialization, *Ascaphus truei*, resolved using two types of genetic markers

**DOI:** 10.1002/ece3.8716

**Published:** 2022-03-18

**Authors:** Cherie M. Mosher, Chris J. Johnson, Brent W. Murray

**Affiliations:** ^1^ 6727 University of Northern British Columbia Prince George British Columbia Canada

**Keywords:** amphibian genetics, *ascaphus*, genotype‐by‐sequencing, microsatellite genotyping, range expansion

## Abstract

Reconstruction of historical relationships between geographic regions within a species’ range can indicate dispersal patterns and help predict future responses to shifts in climate. *Ascaphus truei* (coastal tailed frog) is an indicator species of the health of forests and perennial streams in the Coastal and Cascade Mountains of the Pacific Northwest of North America. We used two genetic techniques—microsatellite and genotype‐by‐sequencing (GBS)—to compare the within‐region genetic diversity of populations near the northern extent of the species’ range (British Columbia, Canada) to two geographic regions in British Columbia and two in Washington, USA, moving toward the core of the range. Allelic richness and heterozygosity declined substantially as latitude increased. The northernmost region had the lowest mean expected heterozygosities for both techniques (microsatellite, *M* = 0.20, SE = 0.080; GBS, *M* = 0.025, *SE* = 0.0010) and the southernmost region had the highest (microsatellite, *M* = 0.88, SE = 0.054; GBS, *M* = 0.20, SE = 0.0029). The northernmost regions (NC and MC) clustered together in population structure models for both genetic techniques. Our discovery of reduced diversity may have important conservation and management implications for population connectivity and the response of *A*. *truei* to climate change.

## Graphical abstract

We used two genetic techniques—microsatellite and genotype‐by‐sequencing (GBS)—to compare the within region genetic diversity of populations near the northern extent of *Ascaphus truei's* range (British Columbia, Canada) to two geographic regions in British Columbia and two in Washington, USA, moving toward the core of the range. We discovered a strong reduction in genetic diversity that may have important conservation and management implications for population connectivity of *A*. *truei*.

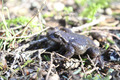

## 1 INTRODUCTION

**FIGURE 1:**
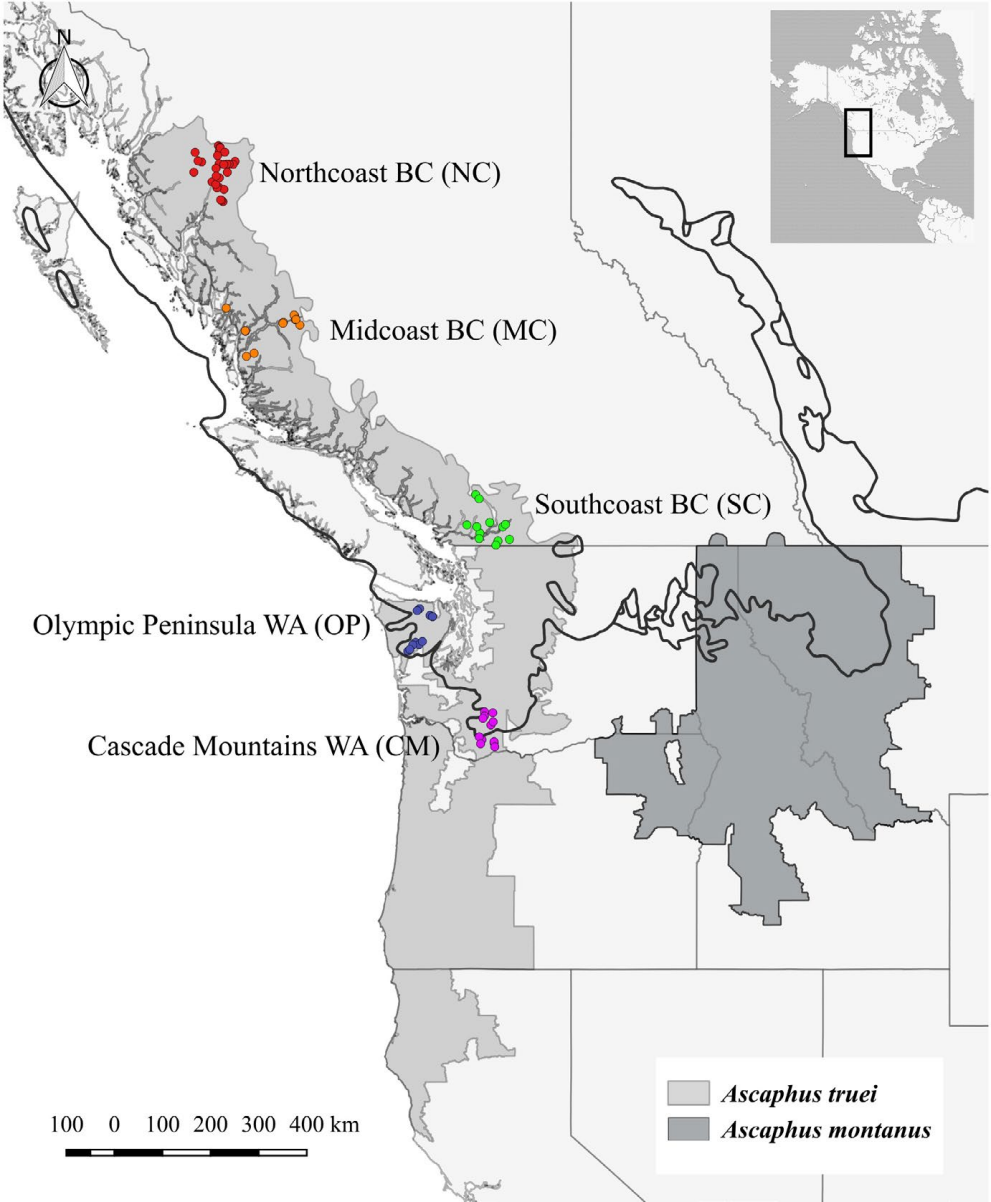
Map of northwestern North America showing the distributions of the two *Ascaphus* species from the International Union for Conservation of Nature and Natural Resources (IUCN, 2015a; IUCN, 2015b), and sampled locations along the northern portion of *Ascaphus truei*'s geographic distribution. “NC” (“Northcoast BC”) sampled stream reaches are red and are the northernmost sampled reaches, “MC” (“Midcoast BC”) stream reaches are orange, “SC” (“Southcoast BC”) stream reaches are green, “OP” (“Olympic Peninsula WA”) stream reaches are in pink, and “CM” (“Cascade Mountains WA”) stream reaches are in blue and are the southernmost sampled reaches. Black line is the extent of ice sheets and ice caps during the Last Glacial Maximum, projected at around ^14^C yr BP, according to Dyke et al. (2003)

Studying populations established following major climatic shifts helps us understand the historical processes that influenced a species’ current geographic distribution. Populations colonized because of dispersal during northern range expansion after the Last Glacial Maximum (LGM), around 25,000 years ago, will likely have experienced more genetic drift than populations in ice‐free habitats, resulting in lower genetic diversity due to founder or bottleneck effects (D'Aoust‐Messier & Lesbarrères, 2015; Eckert et al., 2008; Sagarin & Gaines, 2002; Shafer et al., 2010). However, in areas colonized from multiple refugia, populations may have higher than expected genetic diversities (Petit et al., 1998). The intensity of genetic drift is contingent on population dynamics, the number of historical refugia, the proximity to refugia, the way in which a species radiates out from refugia, and the founding population size (Brunsfeld et al., 2001; Dudaniec et al., 2012). In this study, we examine the genetic diversity of *Ascaphus truei*, the coastal tailed frog, in previously glaciated geographic regions including the northern extent of its range over 1500 km north of the southern fringe of the LGM (Figure 1).

The distribution of amphibians in Canada is greatly influenced by the Pleistocene glaciations (from about 2.5 Mya to 11,000 years ago); most amphibians likely migrated north into the country after the LGM. Longitudinally, British Columbia, Canada, is partitioned into the Cascade and Coast Mountains in the west, the Northern Rocky Mountains in the east, and the Central Interior in between. Several studies have compared the genetic relatedness of amphibian species in the Cascade and Coast Mountains to species in the Rocky Mountains, incidentally providing information on the genetic diversity of those species that have dispersed north of the southern periphery of the LGM (Brunsfeld et al., 2001; Carstens & Richards, 2007; Carstens et al., 2004; Funk et al., 2008; Goebel et al., 2009; Nielson et al., 2001, 2006; Ripplinger & Wagner, 2004). Studies on the northern range expansion of amphibians in western North America were often focused in and near the United States or used low‐resolution genetic markers (Dudaniec et al., 2012; Goebel et al., 2009; Kuchta & Tan, 2005; Metzger et al., 2015; Nielson et al., 2006; Pelletier et al., 2015; Pelletier & Carstens, 2016; Recuero et al., 2006; Ripplinger & Wagner, 2004). The studies that have included geographic regions well north of the southern fringe of the LGM often focused on species with broad distributions or variable habitat requirements (D'Aoust‐Messier & Lesbarrères, 2015; Goebel et al., 2009; Lee‐Yaw & Irwin, 2012; Lee‐Yaw et al., 2008).

For *A*. *truei*, usable habitat during northern expansion may have been narrow longitudinally in some locations, potentially limiting the scope of radiation from its refugia as suitable habitat became available. *A*. *truei* has a narrow geographic range and high habitat specialization (Hayes et al., 2015; Figure 1); even so, its range in Canada extends to Nisga'a Lands ending close to Lisims (the “Nass River”; Figure 1). The longitudinal extent of their range is often limited by appropriate mountain habitat (Dupuis & Friele, 2003), while the latitudinal limits are related to their physiology (i.e., thermal minimum and maximum) and the availability of perennial streams (Bury, 1968; Dupuis & Friele, 2003).

Previous research on the genetic variation of *A*. *truei* was narrow in geographic focus or used low‐resolution marker systems. Ritland et al. (2000) clustered Randomly amplified polymorphic DNA (RAPD)‐based genotypes of *A*. *truei* in British Columbia, Canada into two major groups with the most northern geographic regions (north and mid) clustering into a single group. RAPD markers were ultimately dropped from genetic research due to their lack of reproducibility among studies, as the quality of DNA template and the competing effects of coamplifying loci affected the merit of the results. Nielson et al. (2006) found significant differentiation between the Olympic Mountains (Washington USA) and the Siskiyou Mountains (California and Oregon, USA) using allozyme and mitochondrial DNA. Their sampling efforts did not include Canada.

Our understanding of the genetic relatedness of many species across their geographic range, including *A*. *truei*, may be enhanced by determining relatedness using multiple techniques that target different regions of nuclear and mitochondrial genomes (Kuchta & Tan, 2005; Lee‐Yaw & Irwin, 2012; Nielson et al., 2006). We used three genetic markers: microsatellite genotyping, GBS using nextRAD‐derived genomic libraries for single‐nucleotide polymorphism (SNP) genotyping, and mtDNA haplotypes isolated from the nextRAD genomic libraries. We also compared the variability of SNP genotypes from the GBS dataset to that of the microsatellite genotypes to determine if the nextRAD method provides greater detail for the within and between geographic region genetic diversity due to the greater amount of data across a broader range of the genome (Hodel et al., 2017). We determined the genetic differentiation within and between regions, and related the genetic diversity of northern populations to populations near the core of the geographic range of *A*. *truei*. This work provides important insights into the relationships of populations across the northern portion of *A*. *truei's* range.

## 2 METHODS

### 2.1 Sampling

*Ascaphus truei* is a long‐lived frog whose larvae remain in cold, fast‐flowing streams for 1–4 years before metamorphosis (Brown, 1990; Bury & Adams, 1999). Larvae use a modified oral disc to cling to the underside of substrate within their natal streams; therefore, we targeted this life stage for tissue collection due to the relative ease of locating them. We employed an opportunistic nonrandom sampling scheme for tissue collection. Streams were included based on accessibility by road while also representing the broad study area. Larvae were caught using a dipnet while flipping over rocks. We targeted several locations along a stream reach to minimize the collection of siblings (~100 m apart; Wahbe & Bunnell, 2001). Tissue samples consisted of skin clipped from the posterior of the tail; we avoided the muscle as much as possible. Larvae were retained in a bucket of stream water until they were comfortably swimming, and any bleeding had subsided. They were returned slightly upstream of their capture location. Tissue samples were preserved in 95% ethanol and stored at −80°C.

Sampling was concentrated in the “Northcoast” (NC) region, around Terrace BC, in 2014. The “Midcoast” (MC) region, around Bella Coola BC, and the “Southcoast” (SC), around Chilliwack BC, were sampled during the summer of 2015 (Figure 1). We received purified DNA from stream reaches in the Olympic National Forest and Olympic National Park, referred to as the “Olympic Peninsula” (OP) region (Figure 1; see Spear & Storfer, 2008). We also received DNA from the area around Mt. St. Helens and the lower Columbia River and referred to these as from the “Cascade Mountain” (CM) region (see Spear et al., 2012). DNA was extracted from tail clips using the DNeasy Blood and Tissue kit (Qiagen, Inc., Toronto, ON) following the manufacturer's instructions.

### 2.2 Laboratory protocols

#### 2.2.1 Microsatellite markers

We used ten polymorphic microsatellite DNA markers for the microsatellite analysis (Spear et al., 2008; Table S1). Each locus has a tetranucleotide repeat motif, reducing the potential for typing errors (primers and PCR conditions described in Spear et al., 2008). PCR thermal cycling included an initial denaturation at 95°C for 15 min, followed by 35 cycles at a locus‐specific annealing temperature for 30 s, an extension at 72°C for 30 s, and a further denaturation at 95°C for 30 s. The cycles were followed by an additional elongation at the annealing temperature for 60 s. One primer per pair was labeled with a fluorescent tag (FAM, PET, or VIC). Four amplicon pools were created based on size and generated multilocus genotypes using fragment analysis with the Applied Biosystems 3130xL (Burlington, ON). We scored microsatellite genotypes with GeneMapper (Applied Biosystems).

#### 2.2.2 nextRAD sequencing

We sent purified DNA for nextRAD library preparation, sequencing, and initial filtering to SNPsaurus, LLC (Eugene, OR). Fifteen samples were sent for sequencing in duplicate and triplicate to determine the efficacy of the genotyping method. SNPsaurus converted genomic DNA into nextRAD genotype‐by‐sequencing (GBS) libraries as in Russello et al. (2015). Genomic DNA was randomly fragmented with Nextera reagent (Illumina, Inc), which also ligates short adapter sequences to the ends of the fragments. The Nextera reaction was scaled for fragmenting 25 ng of genomic DNA, although 50 ng of genomic DNA was used for input to compensate for any degraded DNA in the samples and to increase fragment sizes.

Fragmented DNA was then amplified for 27 cycles with 74°C extension, with one of the primers matching the adapter and extending 10 nucleotides into the genomic DNA with the selective sequence GTGTAGAGCC. Thus, only fragments starting with a sequence that could be hybridized by the selective sequence of the primer were efficiently amplified. SNPsaurus sequenced the nextRAD libraries on a HiSeq 4000 with two lanes of 150 base pair (bp), single reads (University of Oregon), and 20x depth of coverage.

### 2.3 Data analyses

#### 2.3.1 Microsatellite markers

In each geographic region, we checked for null alleles and allelic dropout using MICRO‐CHECKER v2.2.3 (van Oosterhout et al., 2004). We tested for linkage disequilibrium between pairs of loci, and for significant deviations from Hardy–Weinberg equilibrium in each region using ARLEQUIN v.3.5.2.2 (Excoffier & Lischer, 2010). Expected heterozygosity (*H_e_*) and observed (*H_o_*) heterozygosity were used to determine the genetic diversity of each region, calculated in POPPR v.2.8.0 for R v.3.4.0 (Kamvar et al., 2014, 2015). We determined the number of repeated microsatellite genotypes per region using GENALEX v.6.5 (Peakall & Smouse, 2006).

#### 2.3.2 nextRAD sequencing

SNPsaurus LLC conducted the bioinformatic analysis of the raw reads to produce a vcf genotype file for population genetic analysis. We used custom scripts that trimmed the sequence reads based on bbduk (BBMAP TOOLS; Bushnell et al., 2017): bash bbmap/bbduk.sh in = $file out = $outfile ktrim = r k = 17 hdist = 1 mink = 8 ref = bbmap/resources/nextera.fa.gz minlen = 100 ow = t qtrim = r trimq = 10. A reference adapter was matched to the reads and all bases to the right were trimmed (as these were single‐end reads), allowing for one mismatch. Reads were trimmed at bases with a quality score of 10 or less and reads shorter than 100 base pairs were removed (Russello et al., 2015). After trimming, an analysis of the reads (bbduk) shows 83.4% of bases had the highest possible quality score and 1.4% had a quality score lower than Q20. Average quality declined slightly along the read length to a minimum of Q37.7 at nucleotide 150.

A de novo reference was created by collecting 10 million reads in total, evenly from the samples, and excluding clusters that had counts fewer than 20 or more than 1000. The remaining clusters were then aligned to each other to identify allelic loci and collapse allelic haplotypes to a single representative. For each sample, all reads were mapped to the reference with an alignment identity threshold of 95% using bbmap (BBMAP TOOLS). Genotype calling was done using SAMTOOLS v1.8 and BCFTOOLS v1.8 (samtools mpileup ‐gu ‐Q 10 ‐t DP, DPR ‐f ref.fasta ‐b samples.txt | bcftools call ‐cv ‐ > genotypes.vcf) (Li, 2011; Li et al., 2009), generating a vcf file.

The vcf file was filtered to remove alleles with a population frequency of less than 3% (referred to as minor allele frequency). Loci were removed that were heterozygous in all samples or had more than 2 alleles in a sample (suggesting collapsed paralogs). The presence of artifacts was checked by counting SNPs at each read nucleotide position and determining that SNP number did not increase with reduced base quality at the end of the read. From the vcf file provided, we removed loci that were variable due to base insertions or deletions using VCFTOOLS v0.1.14 (Danecek et al., 2011).

#### 2.3.3 nextRAD triplicate comparison

We generated a final SNP dataset with all samples from the three British Columbia regions (SC, MC, and NC), including those genotypes in triplicate and duplicate. We expected to have some reduction of genetic diversity in the northern geographic regions and were concerned about genotype call errors, missing data, and paralogous loci in the nextRAD dataset presenting as genetic diversity in these geographic regions (Hodel et al., 2017). We compared the genetic distance between nonreplicated genotypes to the genetic distance of replicated ones. Per geographic region, we converted the genotypes into a pairwise individual‐by‐individual genetic distance matrix for codominant data, with interpolated missing data, using GENALEX (based on simple mismatch distance; Kosman & Leonard, 2005). Per geographic region, we compared mean and 95% confidence intervals of within triplicate genetic distances against the mean and 95% confidence interval for nontriplicate genetic distances. We also created a neighbor‐joining phylogeny using MEGA7 (Kumar et al., 2016) to characterize the relatedness of replicates compared to nonreplicates across all three geographic regions in British Columbia.

#### 2.3.4 Genetic structure

To create a final dataset for analysis of genetic structure, we randomly retained one of the triplicates or duplicates. Text files were generated from the filtered vcf file for additional per region filtering, as the heterozygosity and levels of missing data varied between regions. We removed loci with ≥40% missing data in at least one geographic region and loci with an excessive heterozygosity p‐value of ≤0.005 per region to further reduce the potential impact of paralogs (GENALEX). We tested for significant deviations from Hardy–Weinberg equilibrium in each geographic region using GENALEX. H_e_ and H_o_ were calculated for the final SNP dataset in POPPR.

We used ADEGENET v2.1.1 package for R v.3.4.0 (Jombart, 2008; Jombart & Ahmed, 2011) to calculate F‐statistics and Nei's estimator of pairwise F_ST_ for both molecular marker datasets (ADEGENET relies on HIERFSTAT v0.04.22 for *F*‐statistics). We used 10,000 bootstraps with a lower and upper quantile for confidence intervals of 0.25 and 0.975 to determine pairwise F_ST_ between sets of geographic regions (boot.ppfst).

A population that experienced a recent bottleneck will likely have a lower effective population size than a population that experienced equilibrium or expansion (Nei & Tajima, 1981). We approximated a population parameter, *Θ*, per geographic region with the microsatellite dataset. We calculated *Θ* using mean allele frequency per microsatellite locus under a stepwise mutation model (Haasl & Payseur, 2010) with PEGAS v0.13 for R (theta.msat; Paradis, 2010).

Population structure was modeled in two ways. We used the Bayesian‐based clustering method STRUCTURE v2.3.4 (Pritchard et al., 2000), and also used a multivariate analysis method (discriminant analysis of principal components; DAPC; Jombart et al., 2010). We used DAPC, in addition to STRUCTURE, as it is free of the Hardy–Weinberg equilibrium assumptions of the more commonly used method. DAPC was conducted in the ADEGENET package.

STRUCTURE assigned individuals to different clusters (*K*) ranging from 2 to 10, without prior sample site location and with admixture. It performed 10 iterations with a burn‐in of 100,000 and a running length of 100,000 steps. We included *K*'s based on Ln *P*(D) and Δ*K* values, scored using STRUCTURE HARVESTER (Earl & von Holdt, 2012; Evanno et al., 2005). As we had multiple runs, we generated consensus alignments of clusters for the top *K*'s using CLUMPAK (Kopelman et al., 2015).

For the DAPC, we used find.clusters in ADEGENET to determine the placement of genotypes within clusters. We used spline interpolation α‐score, also in ADEGENET, to determine the number of principal components to retain. All discriminant functions were retained (*N* = 4).

#### 2.3.5 Phylogenetics using mtDNA

We used the previously published *Ascaphus* mitochondrial genome from GenBank (Gissi et al., 2006) with the nextRAD sequencing data to extract mtDNA reads. The mtDNA genome was indexed, each genotype was separated into individual files, and each file was aligned to the mtDNA genome with the Burrows–Wheeler Aligner algorithm BWA‐MEM (BWA; Li & Durbin, 2010). Using SAMTOOLS v1.8, alignments were removed if they had an MAPQ score (−10 log_10_Pr {mapping position is wrong}) of ≤30.

We removed duplicate sequences and merged files using PICARD, retaining a single sample from those sent in triplicate or duplicates for sequencing. Files were sorted and indexed using SAMTOOLS. SNPs were called using FREEBAYES v0.9.10 (Garrison & Marth, 2012). The ploidy was set to 1, and the defaults were used for all other settings. Loci that had more than 5% missing calls across haplotypes were not included in the analysis, and haplotypes with greater than 40% missing calls were removed. We generated a phylogenetic, minimum spanning network using POPART v1.7 (Bandelt et al., 1999; Leigh & Bryant, 2015).

## 3 RESULTS

### 3.1 Microsatellite markers

For the microsatellite analysis, we analyzed 48 genotypes from each of the 5 geographic regions. We used 4 *A*. *truei* larval samples from 12 streams per geographic region (Table S2). Locus A13 showed evidence for a null allele and significant deviation from Hardy–Weinberg equilibrium and was dropped from the final analysis (microsatellite loci = 9). No linkage disequilibrium was found between pairs of loci within each region.

Four microsatellite genotypes in the NC geographic region were shared by more than 1 sample. Three samples shared Genotype 1, two samples shared Genotype 2, four samples shared Genotype 3, and three samples shared Genotype 4 (Figure S3). Only two of the four matching genotypes came from individuals collected in the same stream reach, while most were found in separate streams, some as great as 60 km apart. Three of the matching genotypes had only one heterozygotic locus (of 9) and one had two heterozygotic loci. No other geographic region had individuals with identical genotypes.

### 3.2 nextRAD sequencing

We included 35 genotypes per geographic region in the nextRAD sequencing analysis. The number of sampled streams varied by region (NC = 27, MC = 12, SC = 11, CM = 12, SC = 12). The number of *A*. *truei* samples per stream also varied by region (Table S1).

De novo assembly and initial filtering of nextRAD sequences produced 8690 polymorphic loci. The dataset was reduced to 8213 loci after we removed SNPs based on insertions and deletions. The number of loci was further reduced to 4249 after we removed loci with excessive heterozygosity and excessive missing data per region. We eliminated additional loci because of significant deviations from Hardy–Weinberg equilibrium in two or more regions, yielding a final dataset of 4228 loci.

### 3.3 nextRAD triplicate comparison

Fifteen samples were processed from library construction to SNP genotyping in triplicate or duplicate; seven samples in triplicate from the NC region and three samples in triplicate and one in duplicate from both the MC and SC geographic regions (Table S4). A phylogeny of the filtered nextRAD dataset (4228 loci) showed triplicates and duplicates as separate monophyletic clusters in the SC region. However, repeat genotypes did not cluster together for the MC and NC regions (Figure S5).

**FIGURE 2:**
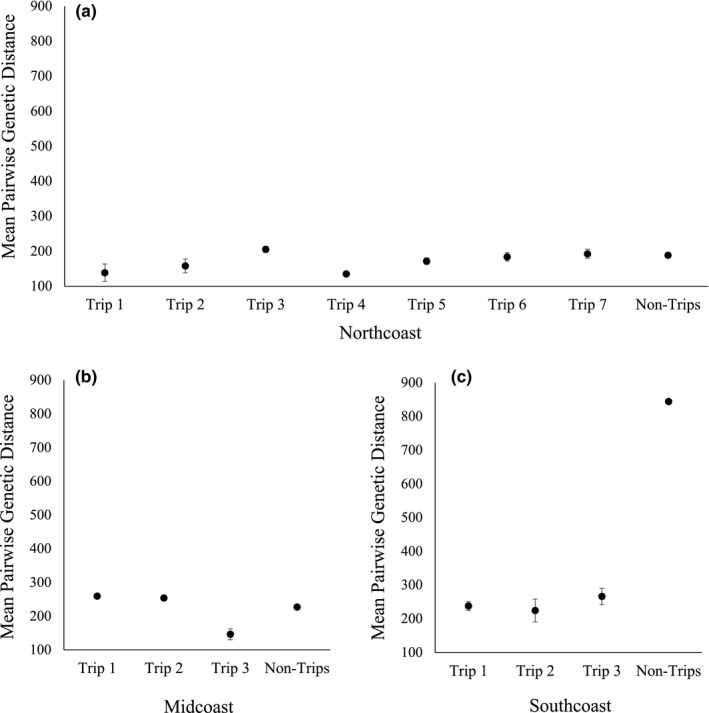
Mean pairwise genetic distance comparison between triplicate and nontriplicate nextRAD genotypes for *Ascaphus truei* in three geographic regions; (a) “Northcoast” (NC) around Terrace, BC, (b) “Midcoast” (MC) around Bella Coola, BC, and (c) “Southcoast” (SC) around Chilliwack, BC. Error bars represent 95% confidence intervals around the mean. “Northcoast” (NC) is the northernmost geographic region in British Columbia, CA and “Southcoast” (SC) is the southernmost. The “Non‐Trips” mean is for all pairwise genetic distance scores that were not associated with a replicated genotype

In the MC and NC regions, mean pairwise genetic distance for triplicates was either greater than or similar to the mean genetic distance for nontriplicates (Figure 2). One triplicate in the NC region (135.67 ± 11.76) and one in the MC region (146.61 ± 31.57) had a lower genetic distance than that of the mean nontriplicates (NC = 188.64 ± 1.05; MC = 227.13 ± 1.92), without overlap in their 95% confidence intervals. In contrast, the pairwise genetic distance for nontriplicates in the SC region (844.65 ± 1.05) was greater than the pairwise distances for the triplicates. We randomly selected and retained one of the replicates for further analysis. Thirty‐five genotypes were randomly selected from each geographic region for a total of 175 genotypes (Table S6).

### 3.4 Genetic variability

For the microsatellite analysis, mean allelic richness across loci provides a measure of genetic variability as loci can have many alleles. The mean allelic richness for 9 loci across geographic regions was 12.27 ± 1.54 and ranged within regions from 3.33 (NC) to 23.56 (CM) (Table 1). Two loci were monomorphic in the NC region and one locus was monomorphic in the MC region. The greatest number of alleles for a locus was sampled from the OP region (43). Mean *H_o_* was 0.60 and ranged from 0.19 (NC) to 0.86 (CM).

**TABLE 1:** Measurements of genetic variation of *Ascaphus truei* by geographic region for microsatellite and nextRAD sequencing data sets

Region	Microsatellite dataset (*N* loci = 9)					nextRAD sequencing dataset (N loci = 4228)			
	*N*	Mean alleles	Mean *H_e_*	Mean *H_o_*	Mean *Θ*	*N*	Alleles	Mean *H_e_*	Mean *H_o_*
OP	48	18.00 (2.43)	0.89 (0.021)	0.84 (0.033)	45.92 (12.78)	35	6828	0.14 (0.0027)	0.14 (0.0027)
CM	48	23.56 (3.63)	0.88 (0.054)	0.86 (0.072)	82.06 (22.68)	35	7229	0.20 (0.0029)	0.18 (0.0027)
SC	48	11.11 (2.62)	0.72 (0.074)	0.70 (0.080)	21.78 (11.50)	35	5898	0.088 (0.0022)	0.079 (0.0021)
MC	48	5.33 (1.56)	0.40 (0.096)	0.39 (0.099)	5.50 (3.81)	35	5223	0.029 (0.0011)	0.033 (0.0012)
NC	48	3.33 (0.91)	0.20 (0.080)	0.19 (0.073)	1.72 (1.31)	35	5101	0.025 (0.0010)	0.028 (0.0011)

Note

*N* is the number of genotypes per geographic region. ‘Mean alleles’ represents mean number of alleles per loci for the microsatellite data and ‘Alleles’ represents total number of alleles across all genotypes for the nextRAD sequencing data. *H_e_* represents expected heterozygosity; *H_o_*, observed heterozygosity. Mean *Θ* is a population parameter based on mean allele frequency per locus for calculating effective population size. Standard errors given in parentheses.

For the nextRAD sequencing, a percentage of polymorphic loci provides a measure of genetic variability as loci have two alleles. NC region had 20.65% polymorphic loci. The MC region had 23.53% polymorphic loci and the SC had 39.50%. The CM region had the highest percent of polymorphic loci (70.98%), followed by the OP region with 61.49%. Mean H_o_ ranged from 0.028 (NC) to 0.18 (CM) (Table 1).

### 3.5 Genetic structure

**FIGURE 3:**
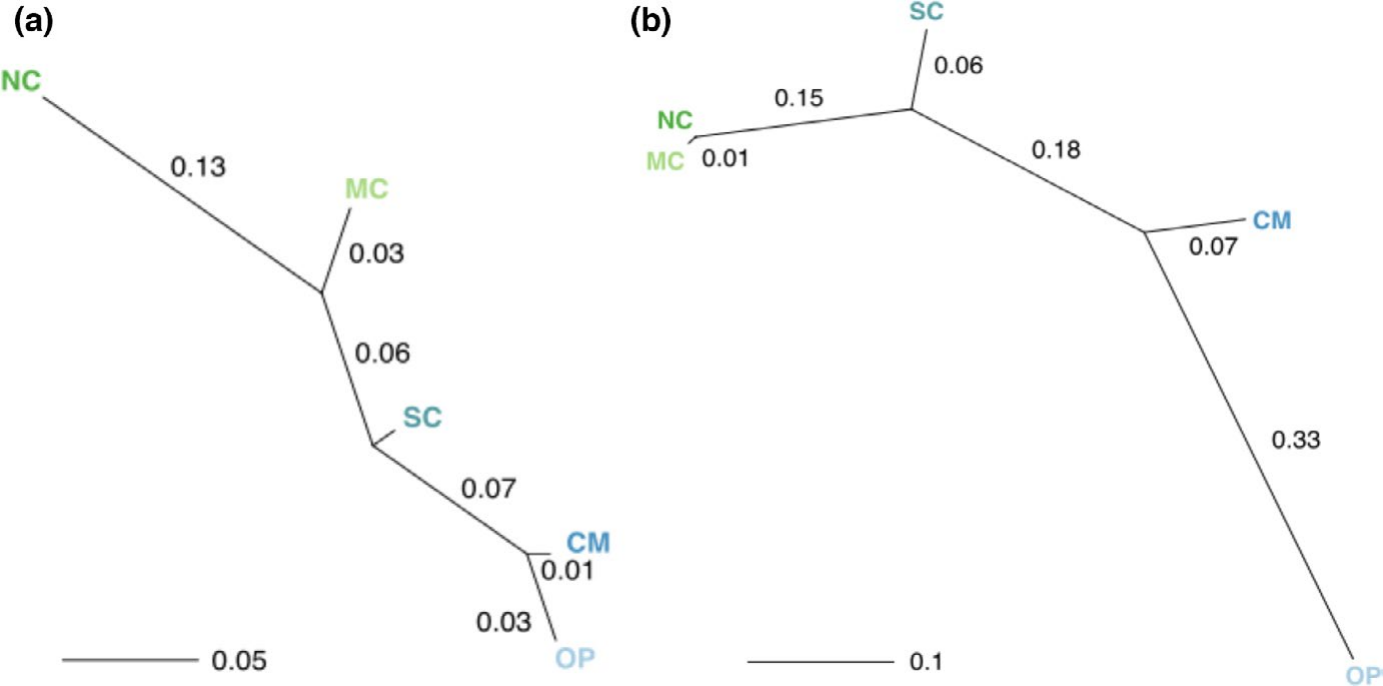
Tree of genetic distance between five geographic regions along the northern half of *Ascaphus truei's* distribution, based on pairwise *F*_ST_, for the (a) microsatellite data and (b) nextRAD sequencing data. “NC” (northcoast) represents the northernmost geographic region, followed by “MC” (midcoast) and “SC” (southcoast). “OP” (Olympic Peninsula) and “CM” (Cascade Mountains) represent the two southernmost regions. Nei's estimator of pairwise F_ST_ and tree construction were performed in ADEGENET

The global F_ST_ was 0.26 for the microsatellites and 0.63 for the nextRAD (Table 2). Pairwise F_ST_ varied between pairs of regions for the microsatellite and nextRAD datasets (Table 3). All pairwise F_ST_ values were significantly different from zero. The genetic distance between the NC and MC geographic regions was largest in the microsatellite dataset (Figure 3). In contrast, the nextRAD dataset had the smallest genetic distance between the NC and MC regions and the largest between the OP and CM.

**TABLE 2:** *F*‐statistics of 5 geographic regions along the northern half of *Ascaphus truei's* range for microsatellite and nextRAD sequencing data

	Microsatellite (9 Loci)	nextRAD (4228 Loci)
*F*_ST_	0.26	0.63
*F*_IT_	0.29	0.66
*F*_IS_	0.043	0.074

**TABLE 3:** Nei's estimator of pairwise *F*_ST_ between pairs of regions for microsatellite and nextRAD sequencing data for *Ascaphus truei* along the northern half of *A*. *truei's* distribution

	Microsatellite (9 Loci)					nextRAD (4228 Loci)			
	OP	CM	SC	MC		OP	CM	SC	MC
CM	0.043*				CM	0.40*			
SC	0.097*	0.067*			SC	0.57*	0.30*		
MC	0.21*	0.18*	0.079*		MC	0.66*	0.41*	0.21*	
NC	0.29*	0.26*	0.19*	0.16*	NC	0.66*	0.40*	0.20*	0.0062*

Note

‘NC’ (northcoast) represents the northernmost geographic region, followed by ‘MC’ (midcoast) and ‘SC’ (southcoast). ‘OP’ (Olympic Peninsula) and ‘CM’ (Cascade Mountains) represent the two southernmost regions. Nei's estimator of pairwise *F*_ST_ was performed in ADEGENET. An * represents a value significantly different of zero using the HIERFSTAT bootstrapping over loci method.

**FIGURE 4:**
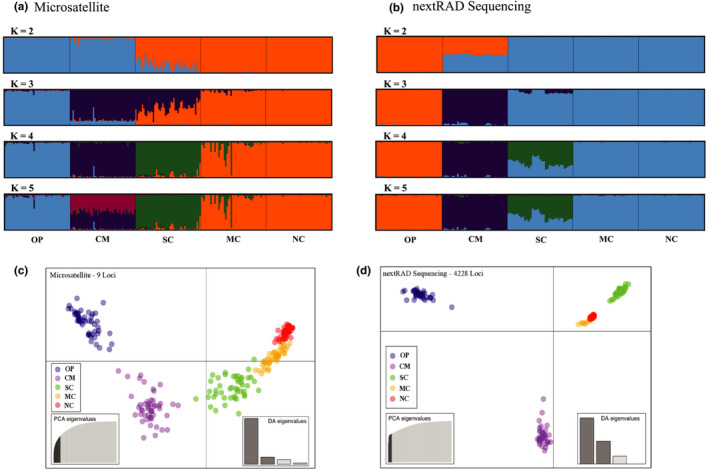
Bayesian STRUCTURE plots (a–b) and plots of discriminant analysis of principal components (DAPC; c–d) based on microsatellite (*N* = 9; a and c) and nextRAD genotypes (*N* = 4228; b and d) for *Ascaphus truei* across five regions in Washington, USA and British Columbia, Canada. STRUCTURE plots represent 2–4 assigned clusters (K) and are partitioned by region. In the DAPC plots, individual genotypes are represented by dots and regions are color coded. “OP” (Olympic Peninsula) and “CM” (Cascade Mountains) represent the two southernmost regions in Washington, USA. “SC” (southcoast), “MC” (midcoast), and “NC” (northcoast) represent geographic regions in British Columbia

The population parameter *Θ*, calculated using the microsatellite dataset, ranged from 82.06 ± 22.68 in the CM region to 1.72 ± 1.31 in the NC region (an almost fiftyfold difference; Table 1). The value of *Θ* decreased from near the core of the geographic range in Washington to the northern edge. In the models of population structure using STRUCTURE, the northernmost regions (NC and MC) clustered together even as the allotted number of clusters increased for both analyses. STRUCTURE plots with clustering from 2 to 5 were included as informative patterns emerged from the data with increasing total clusters (Figure 4). Four clusters (*K* = 4) had the highest Δ*K* and Ln *P*(D) values for the microsatellite analysis (Figure S8), with NC and MC clustering together (Figure 4). The results for nextRAD were more inconsistent, as the Δ*K* value suggested two clusters and the Ln *P*(D) suggested four (Figure S9).

For DAPC plots, we retained 10 principal components for the microsatellite dataset and 8 for the nextRAD dataset (Figure S10). DAPC plots showed similar trends between the two datasets (Figure 4). OP and CM were distinct from each other and from the British Columbia regions. Genotypes from the northern regions (MC and NC) clustered more tightly together than those from the more southern regions in both DAPC plots, and showed little distinction between each other.

### 3.6 mtDNA haplotypes with nextRAD data

**FIGURE 5:**
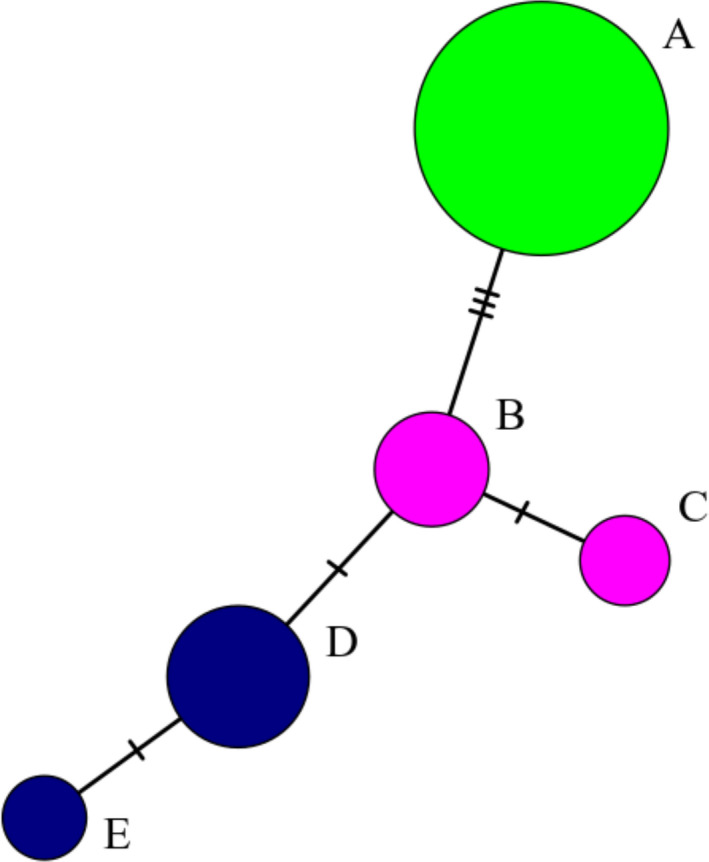
Minimum spanning network for mtDNA from five geographic regions along the northern half of *Ascaphus truei*'s range. The haplotype from British Columbia, Canada is in green (representing three geographic regions). Haplotypes from Olympic Peninsula, USA are in pink and the area surrounding Mt. St. Helens, USA are in blue

Initially, the mtDNA dataset included 36 individuals from the CM geographic region, 48 from the OP region, 44 from SC, 48 from MC, and 48 from the NC region. We used additional samples per geographic region, as compared to the balanced GBS sample design, due to the likelihood of missing data in the mtDNA analysis. After aligning reads from the nextRAD sequencing data with the known mitochondrial genome and filtering, 256 characters were used to define the haplotypes. Several individuals were excluded from the minimum spanning network of mtDNA haplotypes due to large amounts of missing data. The final dataset included 27 individuals from the CM geographic region, 21 from the OP region, 20 from the SC region, 19 from MC, and 25 from NC. The data had six parsimony‐informative sites, a nucleotide diversity of *π* = 0.10, and five haplotypes (A‐E; Figure 5). One haplotype (A) was the only one found in all three BC regions (NC, MC, and SC). The OP region had two haplotypes (B and C; 13 and 8 individuals, respectively) and the CM region had two haplotypes (D and E; 20 and 7 individuals, respectively).

## 4 DISCUSSION

The results of this study provide new insights into the northern expansion of an amphibian species with habitat specialization. Though we expected to find a reduction in genetic diversity in the northernmost geographic regions, the sheer level of reduced diversity was unexpected. Additionally, this study provided new information on GBS with a nontarget species. Our results have implications for future molecular marker selection when researching species with low genetic diversities, and the conservation and management of *A*. *truei* along the northern portion of its range.

### 4.1 Phylogeography

As expected, there was reduced genetic diversity along the northern extent of *A*. *truei's* range as compared to geographic regions more central in its range. We found a single mtDNA haplotype throughout British Columbia. This haplotype is likely the same as the “Zed” haplotype sequenced in northern Washington (Nielson et al., 2006) and northern BC samples (Murray, B. W., *personal communication*). The low number of mtDNA haplotypes throughout geographic regions sampled in the north suggests the combined effect of limited dispersal ability and a single refugium, or a single founding population recolonizing the Coast Mountains following the LGM (Shafer et al., 2010).

This is shown in mtDNA and allozyme genetic markers of other amphibian species (Cortázar‐Chinarro et al., 2017; Eckert et al., 2008; Funk et al., 2008; Goebel et al., 2009; Metzger et al., 2015; Pelletier et al., 2011). In the Pacific Northwest of North America, those species restricted to the Coast and Cascade Mountains had fewer mtDNA and allozyme haplotypes in their northern populations than species with broader ranges (Brunsfeld et al., 2001; Kuchta & Tan, 2005; Steele & Storfer, 2006). For example, *Taricha granulosa* is a new species whose range follows the northwestern coast of North America. Kuchta and Tan (2005) found one allozyme group from Washington, USA to Alaska, USA and a single mtDNA haplotype from northern California, USA to Alaska (with a single satellite haplotype in Washington, USA).

The degree of diversity in more variable, genomic DNA is not reflected in mtDNA and allozyme markers, as they have low mutation rates (McCartney‐Melstad et al., 2018). We found an unexpectedly dramatic reduction in genomic DNA diversity along the northern half of *A*. *truei's* range compared to the core of its geographic scope. The estimated population parameter *Θ* for the southernmost region was almost fiftyfold that of the northernmost region, suggesting genetic drift during the establishment of the northern populations in British Columbia (Kimmel et al., 1998). These results reiterate the likelihood of a northern range expansion following the Pleistocene (~10,000 years ago), most likely from a single refugium. Future research is needed to determine if the extremely low genetic diversity is due to a founder effect from low dispersal rates, a bottleneck effect due to a partial barrier somewhere between the southcoast region (Chilliwack BC) and midcoast region (Bella Coola BC), or other influences on dispersing frogs not yet known.

There may have been at least two refugia for *A*. *truei* during the Pleistocene glaciations; one located in the Klamath‐Siskiyou Mountains (Nielson et al., 2001, 2006; Shafer et al., 2010) and one in the Columbia River area of Washington, USA. Our results may reflect a post‐Pleistocene northern expansion from a Columbia River refugium similar to that described in Steele and Storfer (2006). Populations in mid‐ to northern British Columbia have low levels of diversity; however, it is striking that the range of *A*. *truei* extends thousands of kilometers north of the LGM. Additionally, it is important to conserve areas at both the center and edge of a geographic distribution, as expansion can come from the edge or center of the distribution. Our southcoast region shows greater genetic diversity with strong relatedness to the other BC regions. Studies that clarify the phylogeographic relationship among populations found across the species’ range could reveal responses to a warming climate and future habitat refugia (Françoso et al., 2019).

### 4.2 Marker comparison

Microsatellite genotypes (i.e., simple sequence repeats) and nextRAD genotypes (i.e., SNPs from across the genome) showed similar spatial genetic patterns for *A*. *truei* along the northern half of its range. The DAPC plots for both molecular markers resembled each other, with genotype clusters of the two northernmost regions having little distinction between them. Both markers showed similar trends; there was one exception, that of the pairwise *F*_ST_ estimates among geographic regions. The greatest genetic distance (*F*_ST_) for the microsatellite markers was between the northcoast and midcoast regions. This was the smallest genetic distance in the nextRAD dataset. Conversely, the smallest distance for the microsatellites was between the Cascade Mountains and Olympic Peninsula regions of Washington, USA; the greatest for the nextRAD markers.

Microsatellite markers have the potential for many alleles per locus (Vieira et al., 2016). For our analysis, the mean number of alleles per locus varied considerably among geographic regions (~3 to ~24). A few new alleles in populations that have extremely low diversity (such as the northcoast and midcoast) may hyper‐inflate the difference between populations. Conversely, a large number of alleles per locus in a population leads to high within‐population differences, limiting the between‐population difference that can be calculated with F‐statistics (Allendorf & Luikart, 2007; Charlesworth, 1998; Jakobsson et al., 2013; Tishkoff et al., 2009).

The dramatic difference in the number of alleles between regions could explain why there was a much greater genetic distance between the northcoast and midcoast regions as compared to the distance between the Olympic Peninsula and Cascade Mountain regions for the microsatellite dataset. Low allelic diversity within and between the two most northern regions amplified their pairwise genetic difference and possibly violated the assumptions of the stepwise mutation models. With SNPs, though there may be issues in relation to the frequency of the most common allele (Jakobsson et al., 2013), the limited number of alleles per loci may provide more accurate estimates of genetic distance when there are extreme differences in genetic diversity among sampled areas.

Although SNP data provide more accurate estimates of between‐population differences, caution should be taken when comparing individuals within populations with low genetic diversity. Manual data handling is nearly impossible with GBS data, and there are several potential biases contained within the data, including genotype call errors, missing data, and paralogous loci (Hodel et al., 2017). Several studies have shown that the impact of these potential biases is minimal in comparison to the information provided by several thousand loci across the genome (Attard et al., 2017; Hodel et al., 2017; McCartney‐Melstad et al., 2018; Zang et al., 2018). However, the degree of genetic variability was so low in our two northern geographic regions that the mean pairwise genotypic distance within triplicates was no different than the distance between nontriplicate, potentially misjudging genotype call errors in the data as genetic variation. This suggests potential limitations in the use of GBS for within‐population studies, that is, analysis of landscape genetics, for populations with extremely low genetic diversity.

We suggest using an additional check, such as analyzing samples in triplicate, if using GBS techniques with species that are known to have or may have extremely low genetic diversity. This study demonstrates there are limits in how fine the geographic scale can be for low diversity species (including many endangered species), where the genetic signal is difficult to differentiate from potential biases in the data. Checking the noise to signal ratio will ensure the accuracy and efficacy of any population, landscape, or spatial genetic study using GBS.

## 5 CONCLUSIONS

New methodologies and technologies in genetic and genomic research may deliver vastly more data; however, they also deliver more noise. Our research shows that GBS requires a check to ensure that the genetic diversity within the studied species is great enough to overpower the noise in the data. However, if operating at the appropriate geographic scale, GBS data provide a more accurate picture of the genetic distance among populations compared to microsatellites.

Our results reflected a northern range expansion into British Columbia following the late Pleistocene glaciations. The northern portion of the range may not reflect the total number of refugium nor *A*. *truei's* recent dispersal ability in the southern and central portions of its range. It is likely that *A*. *truei* had a very low dispersal ability during northern range expansion yet their range extends far north of the LGM, around 25,000 years ago. The long‐term implications of the dramatic reduction in genetic diversity are not known; however, we recommend that managers consider the potential implications considering ongoing climate changes.

## AUTHOR CONTRIBUTIONS

**Cherie M. Mosher:** Data curation (lead); Formal analysis (lead); Investigation (lead); Methodology (lead); Visualization (lead); Writing – original draft (lead). **Chris J. Johnson:** Conceptualization (lead); Funding acquisition (lead); Investigation (supporting); Methodology (supporting); Supervision (lead); Validation (supporting); Writing – review & editing (lead). **Brent W. Murray:** Conceptualization (lead); Data curation (supporting); Formal analysis (supporting); Funding acquisition (lead); Investigation (supporting); Methodology (supporting); Supervision (lead); Visualization (supporting); Writing – review & editing (lead).

## Supporting information

Appendix S1

Table S1, S2, S4, S6

## Data Availability

The data that support the findings of this study are openly available in Dryad at https://doi.org/10.5061/dryad.37pvmcvn0. We have included genotype datasets, both microsatellite and nextRAD, in the [Link], [Link] for online publication. We will make our code available and will provide it upon request.
