# Supplementary material for: Reduced genetic diversity associated with the northern expansion of an amphibian species with high habitat specialization, Ascaphus truei, resolved using two types of genetic markers
Source: Ecol Evol. 2022 Mar 18;12(3):e8716. doi: 10.1002/ece3.8716 (PMC8931771; doi:10.1002/ece3.8716)
Supplement: Supplementary file 1 — Appendix S1 [file ECE3-12-e8716-s001.docx]

**Supplemental Information for:**

Reduced genetic diversity associated with the northern expansion of an amphibian species with high habitat-specialization, Ascaphus truei, resolved using two types of genetic markers

Dr. Cherie Mosher, Dr. Chris Johnson, Dr. Brent Murray

**Table of Contents for Figures:**

| **Figure S3** | Page 3 |
| --- | --- |
| **Figure S5** | Page 4 |
| **Figure S8** | Page 5 |
| **Figure S9** | Page 6 |
| **Figure S10** | Page 7 |
| **Figure S11** | Page 8 |

**Table of Contents for Corresponding Tables in Excel Spreadsheet*:**

| **Table S1** | Sheet Sup Table S1 |
| --- | --- |
| **Table S2** | Sheet Sup Table S2 |
| **Table S4** | Sheet Sup Table S4 |
| **Table S6** | Sheet Sup Table S6 |

***Legends for tables are on Page 2 of this document.**

**Legends for supplemental tables**

**Table S1.** Forward and reverse primer sequences, annealing temperature (T*_A_*), allelic size ranges, and GenBank Accession numbers for 10 microsatellite loci for *A. truei* (from Spear et al., 2008). The fluorescent dye label for each forward primer is indicated before the sequence. PCR included 1 μl of purified DNA, 0.1 μl of the forward primer (20 nM), 0.2 μl of a fluorescent tag, 0.2 μl of the reverse primer (20 nM), 3.5 μl of RNase-free H_2_O, and 5 μl of multiplex PCR master mix (Qiagen, Inc., Toronto, ON).

**Table S2**. Genotypes of 240 *A. truei* from 5 geographic regions based on 9 microsatellite loci. ‘Pop ID’ is the code for the geographic region. The two alleles per locus are recorded in separate columns.

**Table S4.** Genotypes of 172 *A. truei* from 3 geographic regions based on 4228 loci with single nucleotide polymorphisms discovered using nextRAD sequencing. Geographic regions are coded by number in the ‘Pop ID’ column. Thirteen samples were sequenced in triplicate and 2 were sequenced in duplicate. The two alleles per locus are recorded in separate columns. Missing data is coded with ‘0’.

**Table S6.** Genotypes of 175 *A. truei* from 5 geographic regions based on 4228 loci with single nucleotide polymorphisms discovered using nextRAD sequencing. Geographic regions are coded by number in the ‘Pop ID’ column. The two alleles per locus are recorded in separate rows. Missing data is coded with ‘-9’.


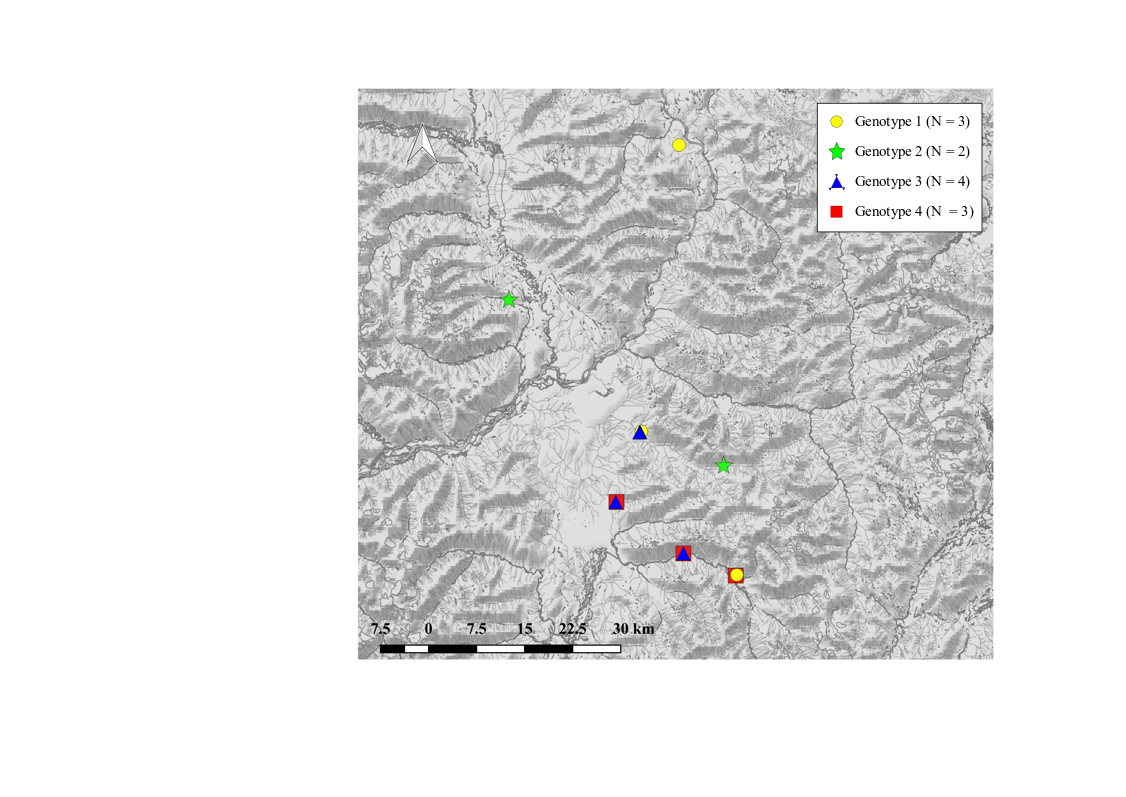


**Figure S3.** Repeated *A. truei* genotypes for 9 microsatellite loci of the 'NC' region (near Terrace BC). Genotype 3 had two of the same genotype from the same stream reach.


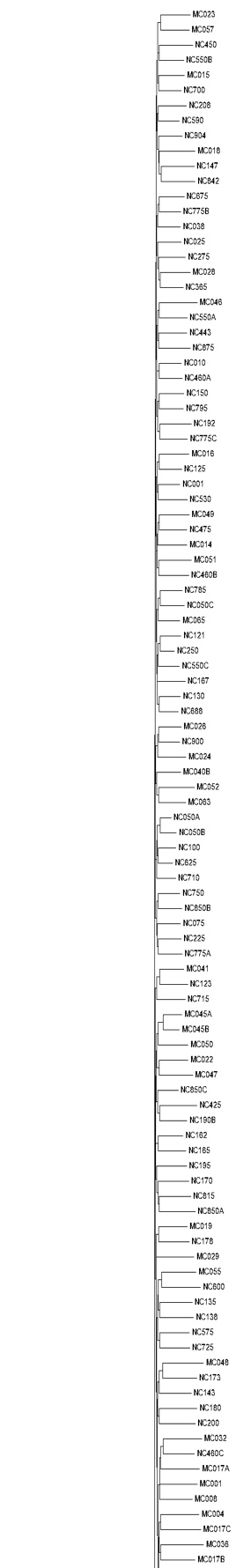

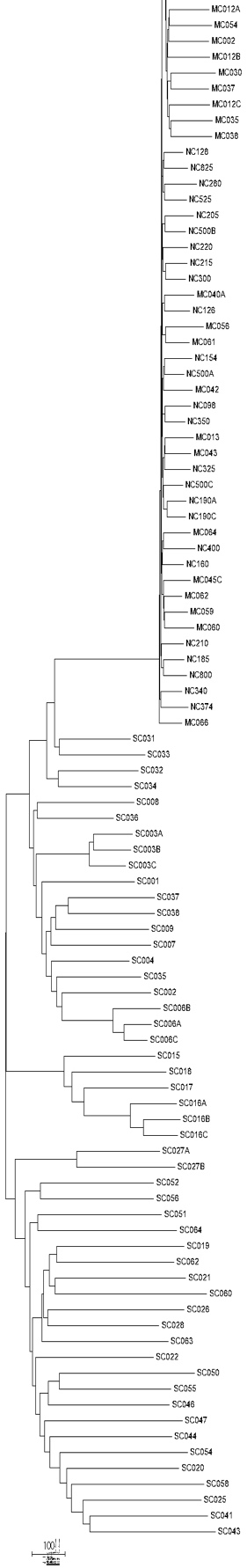


**Figure S5**. Phylogeny of triplicate and non-triplicate nextRAD genotypes for *A. truei* in three geographic regions in B.C. 'NC' designates genotypes from around Terrace, BC, 'MC' from around Bella Coola, BC, and 'SC' around Chilliwack, BC. Triplicates have the same genotype identification and are distinguished by an 'A', 'B', or 'C'.


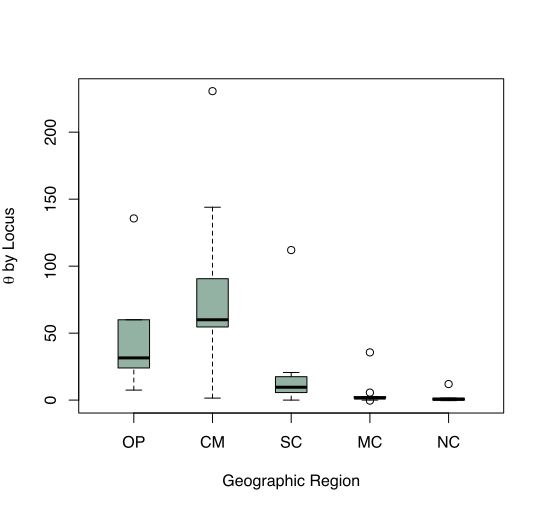


**Figure S8.** Box plots of a population parameter, *Θ*, calculated using allele frequency per microsatellite locus under a stepwise mutation model for 5 geographic regions along the northern half of *A. truei's* distribution. The black line is the median, upper and lower limits of the box are the 75th and 25th percentiles, respectively, the whiskers extend up to 1.5 times the interquartile range, and outliers are represented as points.


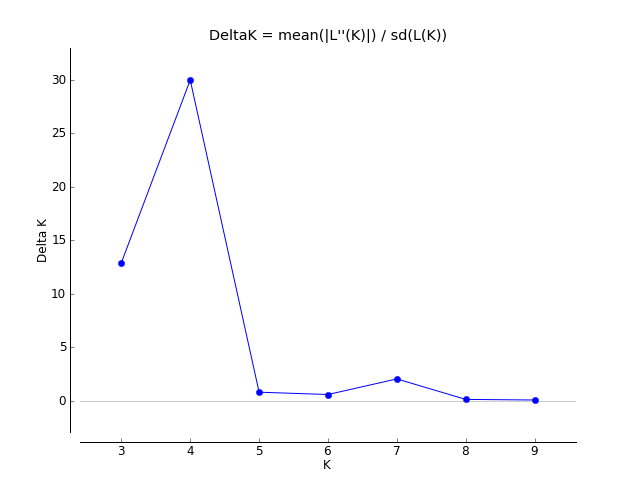

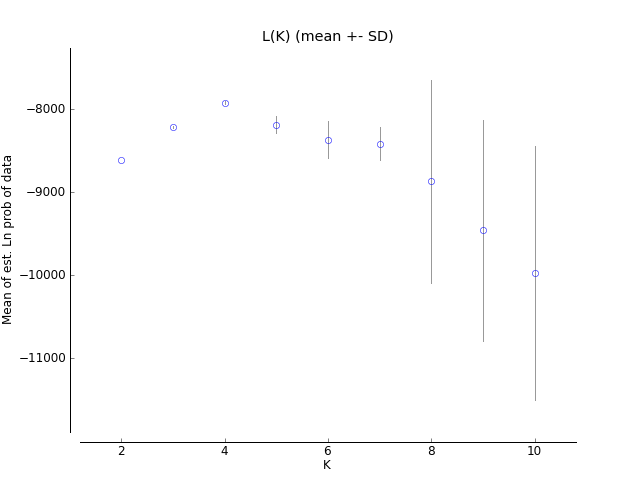


**Figure S9**. The delta *K* and Ln *P*(D) values from the program STRUCTURE and compiled by the program STRUCTURE HARVESTER for microsatellite genotypes of *A. truei* from 5 geographic regions along the northern half of its distribution. Genotypic clusters (K) ranged from 2 to 10.

**Figure S9**. The delta *K* and Ln *P*(D) values from the program STRUCTURE and compiled by the program STRUCTURE HARVESTER for nextRAD sequencing genotypes of *A. truei* from 5 geographic regions along the northern half of its distribution. Genotypic clusters (K) ranged from 2 to 10.


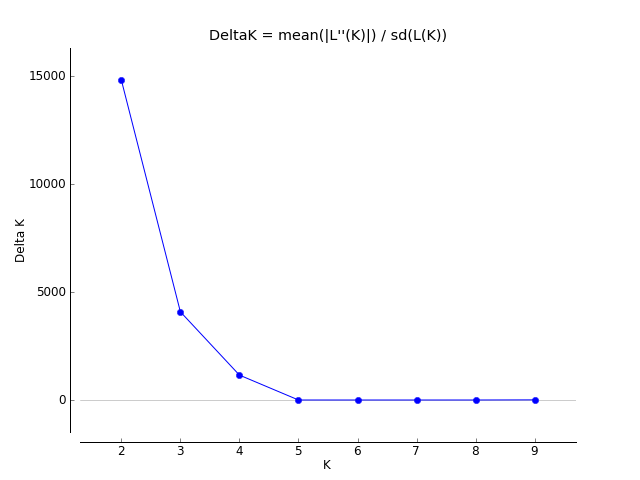

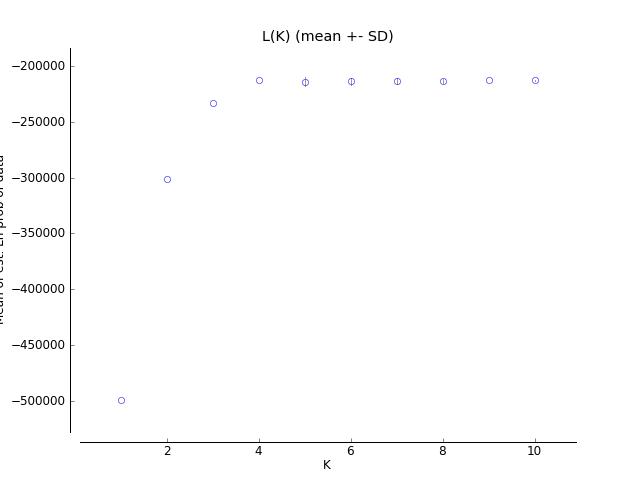


**Figure S10**. The delta *K* and Ln *P*(D) values from the program STRUCTURE and compiled by the program STRUCTURE HARVESTER for genotype-by-sequencing genotypes of *A. truei* from 5 geographic regions along the northern half of its distribution. Genotypic clusters (K) ranged from 2 to 10.


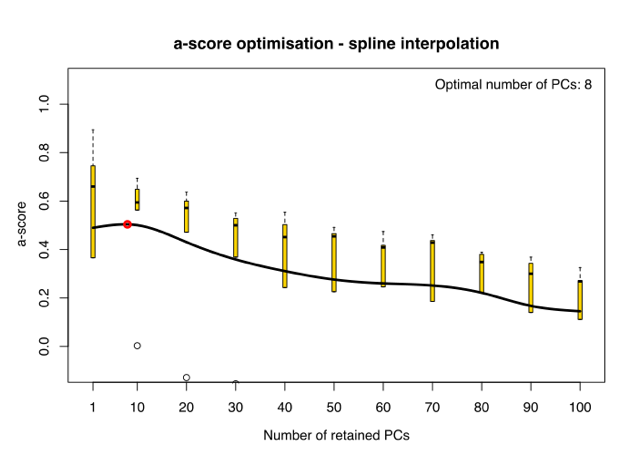

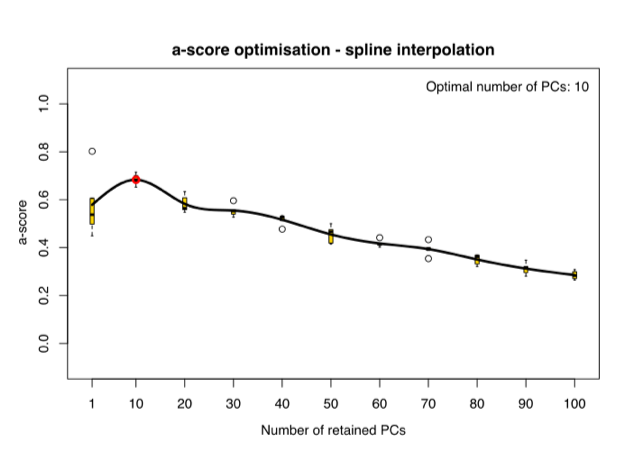


**Figure S11.** The a-scores of principle components for (a) microsatellite genotypes and (b) nextRAD sequencing genotypes for *A. truei* across the northern half of its range. The red circle represents the optimal number of PCs to retain for discriminant analysis of principle components.
